# Remyelination in humans due to a retinoid‐X receptor agonist is age‐dependent

**DOI:** 10.1002/acn3.51595

**Published:** 2022-05-19

**Authors:** Christopher E. McMurran, Trisha Mukherjee, J William L. Brown, Andrew W. Michell, Declan T. Chard, Robin J. M. Franklin, Alasdair J. Coles, Nick G. Cunniffe

**Affiliations:** ^1^ Department of Clinical Neurosciences University of Cambridge Cambridge UK; ^2^ NMR Research Unit, Queen Square Multiple Sclerosis Centre, Department of Neuroinflammation University College London (UCL) Queen Square Institute of Neurology London UK; ^3^ Clinical Outcomes Research Unit (CORe) University of Melbourne Melbourne Australia; ^4^ National Institute for Health Research (NIHR) University College London Hospitals (UCLH) Biomedical Research Centre UK; ^5^ Wellcome‐MRC Cambridge Stem Cell Institute University of Cambridge Cambridge UK

## Abstract

Remyelination efficiency declines with advancing age in animal models, but this has been harder to demonstrate in people with multiple sclerosis. We show that bexarotene, a putatively remyelinating retinoid‐X receptor agonist, shortened the visual evoked potential latency in patients with chronic optic neuropathy aged under 42 years only (with the effect diminishing by 0.45 ms per year of age); and increased the magnetization transfer ratio of deep gray matter lesions in those under 43 years only. Addressing this age‐related decline in human remyelination capacity will be an important step in the development of remyelinating therapies that work across the lifespan.

## Introduction

Therapeutic enhancement of remyelination is a promising strategy to prevent axonal degeneration and related progressive disability in people with multiple sclerosis (MS). In animal models, remyelination becomes inefficient with advancing age, due to both an intrinsic decline in oligodendrocyte progenitor cells (OPCs)^1^ and adverse changes in their environment.^2^, ^3^ Much preclinical research is therefore focused on interventions to reverse hallmarks of aging in OPCs––for example, using rejuvenating drugs, dietary interventions or gene editing––to mitigate the age‐related decline in remyelination (reviewed [^4^]).

With putatively remyelinating drugs reaching clinical trials, the effect of patient age on therapeutic remyelination in humans can now be explored.^5^ Activation of retinoid‐X receptor (RXR) signaling can promote remyelination by stimulating OPC differentiation and clearance of myelin debris.^6^, ^7^ In a two‐center, randomized, double‐blind, placebo‐controlled, phase 2a trial, we recently showed that bexarotene, an RXR agonist, led to statistically significant improvements in the latency of the full‐field visual evoked potential (VEP), and of the MTR of gray matter (GM) lesions (deep and cortical) and brainstem lesions.^8^ We hypothesized that the response to bexarotene would decline with age, and so investigated the effect of patient age on markers of remyelination in a post hoc analysis of the CCMR One trial (ISRCTN14265371).

## Methods

The full protocol and results from the CCMR One trial are published elsewhere.^8^ Briefly, patients with relapsing remitting MS aged 18 to 50 with Expanded Disability Status Scale (EDSS) 0.0 to 6.0 were recruited at centers in Cambridge and Edinburgh. Participants were randomized to receive either 300 mg/m^2^ (up to 750 mg) per day oral bexarotene (*n* = 26) or equivalent placebo tablets (*n* = 26). All participants underwent full‐field VEP testing and MRI brain at baseline and 6 months. Data were analyzed by intention to treat all those completing baseline and 6‐month assessments (*n* = 49). The age range of patients receiving bexarotene was 29 to 49 (mean 40.4) and the placebo group 25 to 49 (mean 38.0).

In the current work, for VEP analysis, the effect of patient age was estimated using linear mixed models for eyes nested within patients, with patient random intercepts. The change in P100 latency was regressed on an interaction between age, treatment group, and baseline value (≤118/>118 ms), as well as three binary minimization factors: EDSS (≤4.0/> 4.0), gender and trial center. Residuals were examined for departures from normality and homoscedasticity, and satisfied assumptions. For MRI analysis, lesions were nested within patients, with patient random intercepts. Change in whole lesion MTR was regressed on an interaction between age, treatment group and lesion location, as well as baseline MTR and the three minimization factors. Residuals for the MTR models were non‐normal, so confidence intervals were verified using a bootstrap approach with 500 replicates. Differences between treatment and control groups as a function of age were calculated using the Johnson–Neyman technique.

## Results

### VEPs

**Figure 1:**
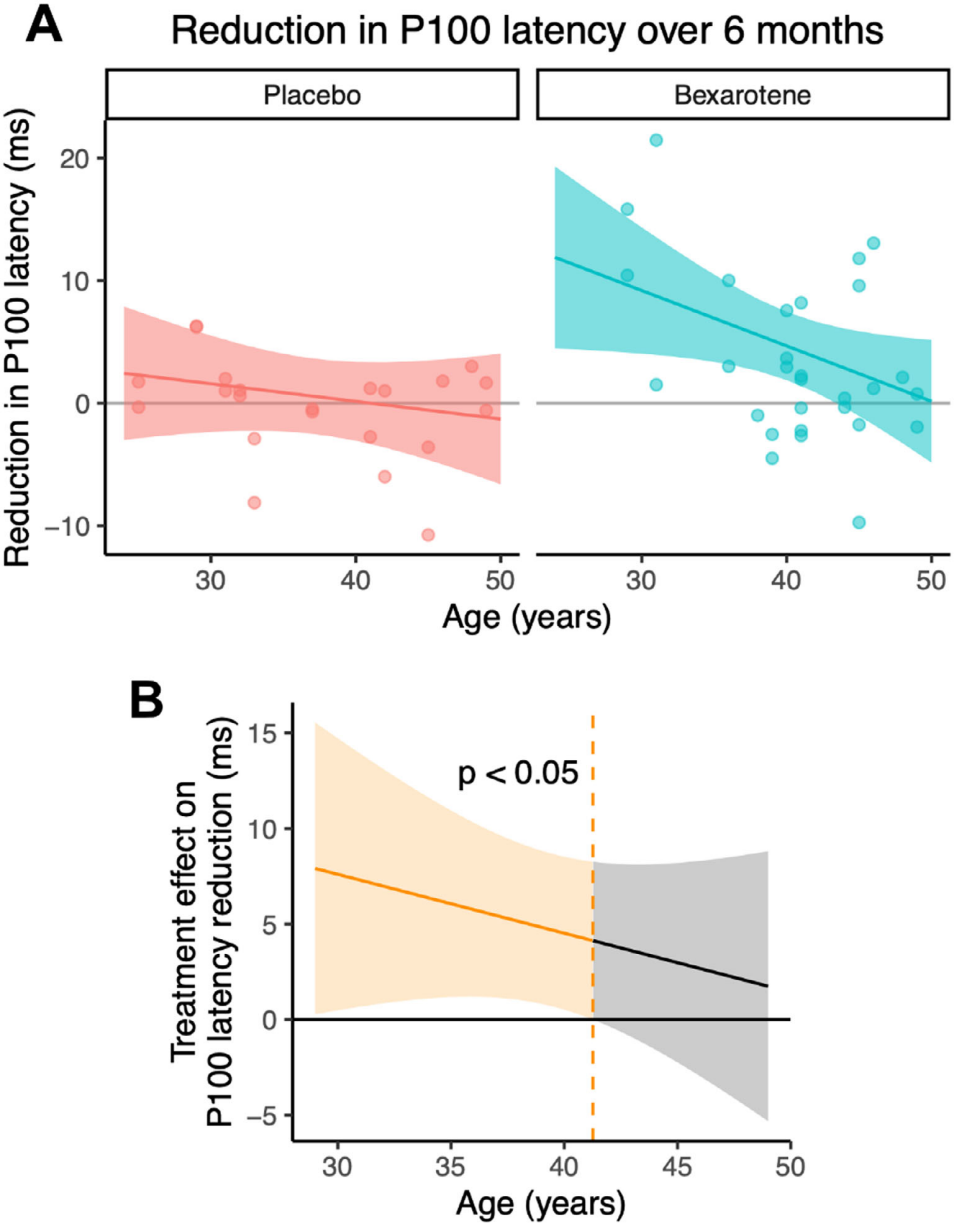
(A) Variation of P100 latency reduction (improvement) with patient age for eyes with a prolonged baseline latency (>118 m). Each data point represents an eye, and the model fit with 95% confidence interval is shown. (B) Treatment effect (bexarotene vs. placebo) from (A) as a function of age. The region to the left of the dashed line shows the age range at which there is a significant treatment effect with type 1 error rate (*α*) = 0.05. [Colour figure can be viewed at wileyonlinelibrary.com]

Previous work has identified the most robust effects of pro‐remyelinating therapies on VEP among eyes with a prolonged baseline P100 latency (>118 ms).^8^, ^9^ Analyzing these eyes from participants of CCMR One (*n* = 51 eyes), bexarotene shortened the P100 latency maximally in younger patients (Fig. 1A). With increasing age, the P100 improvement among patients on bexarotene diminished by 0.45 ms/year (95% CI 0.03 to 0.88 ms/year; *p* = 0.044). Compared to patients on placebo, bexarotene significantly improved P100 latency only up to the age of 42 years (*α* = 0.05, Fig. 1B). Ten patients (63%) of those with prolonged baseline latency in at least one eye receiving bexarotene were younger than this age. The age‐dependence of P100 improvement in the bexarotene group was magnified when eyes affected by optic neuritis during the trial or in the previous 5 years were excluded (reduction by 0.64 ms/year; 95% CI 0.24 to 1.04 ms/year; *p* = 0.004; *n* = 42 eyes).

To investigate whether this age‐dependence may represent an effect of disease duration, we replaced the age term in our model with disease duration (years since first MS symptom onset). Unlike age, disease duration did not significantly modify the P100 improvement in the bexarotene group (reduction by 0.18 ms/year; 95% CI: −0.26 to 0.62 ms/year; *p* = 0.43). We also considered whether older patients might have fewer surviving axons by analyzing baseline VEP amplitude. Age did not significantly reduce baseline amplitude among those eyes with prolonged baseline latency (reduction of 0.11 μV/year; 95% CI −0.09 to 0.31 μV/year; *p* = 0.29), but when all eyes were included, there was a borderline significant age‐dependent reduction in amplitude (by 0.16 μV/year; 95% CI 0.00 to 0.33 μV/year; *p* = 0.059).

### MRI

**Figure 2:**
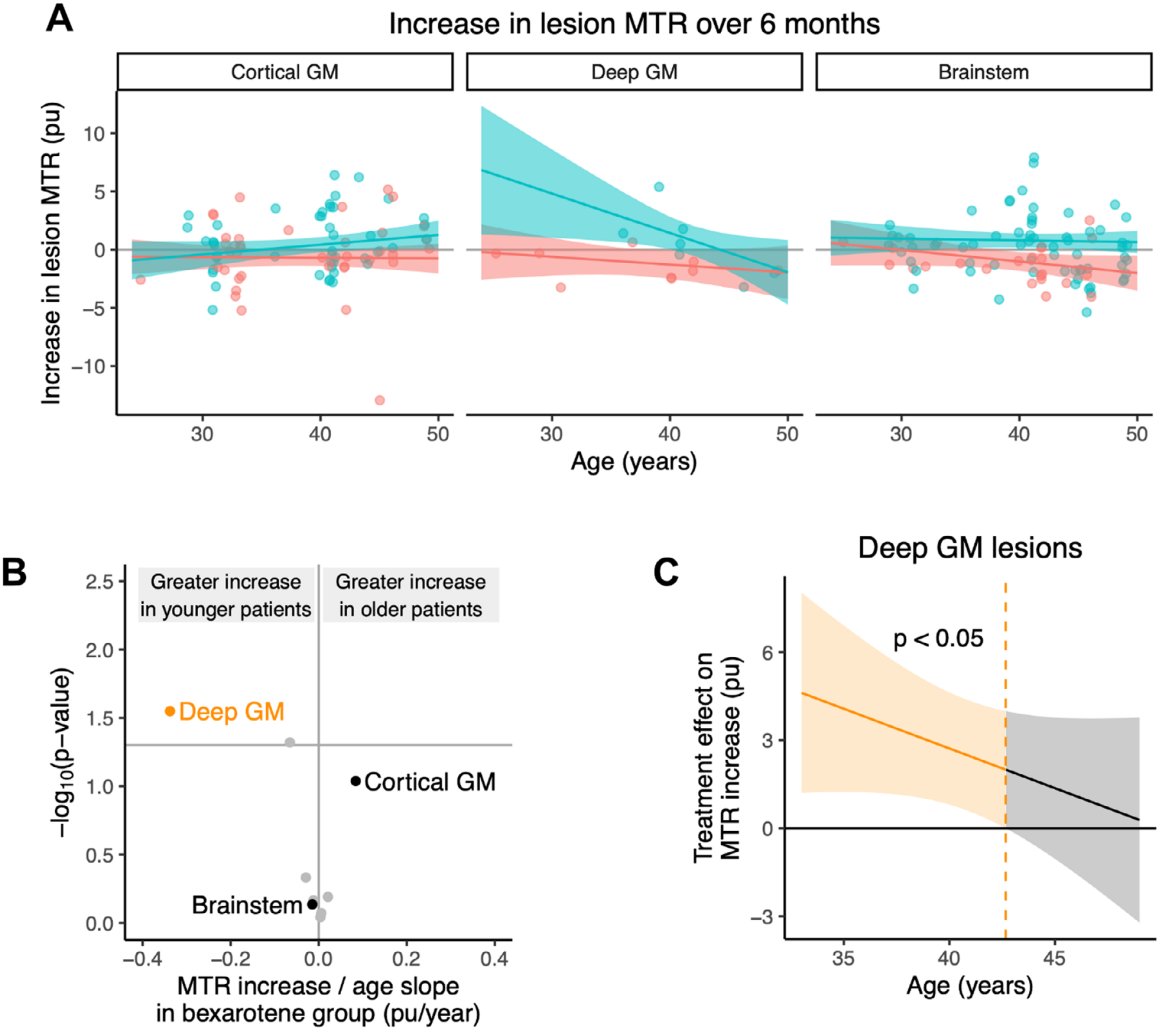
(A) Variation of lesion MTR increase (improvement) with patient age for the three regions with statistically significant increases in MTR.^8^ The bexarotene group is shown in blue and placebo in red. Each data point represents a lesion, with superimposed horizontal jitter to better visualize lesions with the same patient age. The model fit with 95% confidence interval is shown. (B) Volcano plot showing the effect of age on lesion MTR improvement within the bexarotene group for the locations plotted in (A). Other brain regions are shown in gray for comparison. (C) Treatment effect (bexarotene vs. placebo) for deep gray matter lesions as a function of age. The region to the left of the dashed line shows the age range at which there is a significant treatment effect with type 1 error rate (*α*) = 0.05. GM = gray matter, MTR = magnetization transfer ratio. [Colour figure can be viewed at wileyonlinelibrary.com]

Bexarotene was previously found to cause a significant increase (improvement) in MTR signal in deep and cortical GM and brainstem lesions.^8^ In the bexarotene group, we identified an age‐related attenuation in MTR increase among deep GM lesions (−0.34 pu/year; 95% CI −0.64 to −0.04 pu/year; *p* = 0.028, *n* = 16 lesions from 14 participants, Fig. 2A and B). Compared to patients on placebo, bexarotene significantly increased deep GM lesion MTR only up to a patient age of 43 years (*α* = 0.05, Fig. 2C). Four patients (67%) of those receiving bexarotene with at least one deep GM lesion were younger than this age threshold. MTR increase in cortical GM lesions (0.08 pu/year; 95% CI −0.01 to 0.18 pu/year; *p* = 0.09; *n* = 85 lesions from 28 participants) and brainstem lesions (−0.01 pu/year; 95% CI −0.10 to 0.07 pu/year; *p* = 0.73; *n* = 88 lesions from 36 participants) did not significantly depend on age in the bexarotene group. In the placebo arm, the magnitude of MTR decrease (worsening) increased with age in both brainstem and deep GM lesions (Fig. 2A) though these age effects did not reach statistical significance.

Outside radiologically identified lesions, baseline MTR trended downwards with increasing age, but this was neither significant in normal‐appearing white matter (−0.02 pu/year; 95% CI −0.07 to 0.03; *p* = 0.36) nor total GM (−0.02 pu/year; 95% CI −0.06 to 0.02; *p* = 0.34). There was no age range within which bexarotene had a significant treatment effect in these non‐lesion areas (*α* = 0.05).

### Toxicity

Bexarotene was poorly tolerated in CCMR One, with common side effects including hypertriglyceridemia, central hypothyroidism, and neutropenia.^8^ We found that the number of adverse events among patients receiving at least one dose of bexarotene was not significantly affected by age (0.017 events/year of age; 95% CI: −0.16 to 0.19 events/year of age; *p* = 0.85).

## Discussion

Here, we demonstrate––for the first time in humans––that the response to a remyelinating drug decreases with age. Bexarotene, an RXR agonist, significantly improved VEP latency and MTR of deep GM lesions only in patients up to their early 40s. This is consistent with rodent models where remyelination fails with increasing age,^1^, ^3^, ^4^ and clinical studies showing age‐dependent accumulation of disability among people with MS.^10^, ^11^, ^12^ Indeed, the fifth decade is typically when patients develop progressive disease, regardless of prior disease course,^13^ giving a possible indication of the timescale of age‐related remyelination failure in humans.

Of note, the picture of age‐dependence in MRI outcomes was less conclusive than that of the VEP results. While remyelination declined with age in deep GM, no decline was seen in cortical GM or brainstem lesions. This heterogeneity might point toward genuine regional differences in the resilience of remyelination at older ages. For example (while not directly compared to deep GM lesions) pathological literature suggests that cortical GM lesions can actively remyelinate in the brains of patients into their 70s.^14^ However, it should be emphasized that our study describes relatively few lesions from deep GM, and further work should employ MRI sequences more sensitive to GM lesions to verify these findings.

The CCMR One trial, which included patients from young adulthood to middle age (25–50), was neither designed nor powered to assess the effect of age on remyelination. Future studies should therefore examine a broader age range to assess remyelination across the human lifespan. We also found that baseline VEP amplitude tended to decline with age. As remyelination requires intact axons, differential axonal survival should be considered as a potential confounding factor. Despite these limitations, converging neurophysiological and MRI evidence indicates that remyelination varies with age in people with MS.

Our findings here contrast somewhat with the RENEW trial, in which the best VEP latency response to opicinumab was seen in patients aged 33 and older.^5^ These differences may reflect our focus on chronic optic neuropathy rather than acute optic neuritis; indeed, the age‐related attenuation in VEP response in our study was strengthened when eyes with recent optic neuritis were excluded. One similarity between the studies was the poor response of older patients receiving placebo, suggesting little baseline remyelination in this group.

Any approach to promoting remyelination in MS might be limited by the intrinsic capacity of the aged CNS to repair. Encouragingly, this capacity can be enhanced in rodent models through interventions that rejuvenate an older animal's biological age, such as exposure to a youthful systemic environment,^3^ intermittent fasting^1^ or drugs including niacin^15^ and metformin.^1^ With the demographic of people with MS getting older,^16^ such interventions are likely to play an important role in emerging strategies to promote remyelination and reduce disability.

## Author Contributions

CEM, AJC, and NGC conceived and designed the study. JWLB, AWM, AJC, and NGC acquired the data. CEM, JWLB, DTC, RJMF, AJC, and NGC analyzed the data. CEM, TM, JWLB, and NGC drafted a significant portion of the manuscript.

## Conflict of Interest

CEM, TM, AWM, AJC, and NGC report no conflict of interest. JWLB reports personal fees from Biogen for real‐world evidence consultation, outside the submitted work. DTC is a consultant for Biogen and Hoffmann‐La Roche. In the last 3 years he has received research funding from Hoffmann‐La Roche, the International Progressive MS Alliance, the MS Society, and the National Institute for Health Research (NIHR) University College London Hospitals (UCLH) Biomedical Research Centre, and speaker's honorarium from Novartis. He co‐supervises a clinical fellowship at the National Hospital for Neurology and Neurosurgery, London, which is supported by Merck. RJMF reports consulting fees from Frequency Therapeutics, Rewind Therapeutics and Biogen.
